# Accumulation of Nucleolar Inorganic Polyphosphate Is a Cellular Response to Cisplatin-Induced Apoptosis

**DOI:** 10.3389/fonc.2019.01410

**Published:** 2019-12-12

**Authors:** Lihan Xie, Asavari Rajpurkar, Ellen Quarles, Nicole Taube, Akash S. Rai, Jake Erba, Benjamin Sliwinski, Moses Markowitz, Ursula Jakob, Daniela Knoefler

**Affiliations:** ^1^Department of Molecular, Cellular, and Developmental Biology, University of Michigan, Ann Arbor, MI, United States; ^2^Johns Hopkins Bloomberg School of Public Health, Baltimore, MD, United States

**Keywords:** cisplatin, polyphosphate, apoptosis, caspases, nucleolus, resistance

## Abstract

The chemotherapeutic drug cisplatin, which targets DNA, serves as one of the main staples in cancer treatment. Yet, the therapeutic application of cisplatin is limited by two major challenges: the occurrence of reversible and irreversible side effects due to non-specific toxicity, and the intrinsic or developing resistance of tumor cells toward cisplatin. Here we demonstrate that cancer cells respond to cisplatin treatment with the nucleolar accumulation of inorganic polyphosphate (polyP), a universally conserved high-energy compound. PolyP accumulation positively correlates with the levels of activated caspase-3, suggesting a novel role of polyP in cisplatin-mediated apoptosis. In support of this finding, we discovered that administration of exogenous polyP increases cisplatin-induced toxicity in select cancer cell lines, raising the exciting possibility that enhancing endogenous polyP levels might be a novel mechanism to sensitize cancer cells to cisplatin treatment.

## Introduction

About 1,600 patients die of cancer each day, making cancer the disease with the second highest mortality rate in the United States (1). Platinum-containing drugs such as cisplatin, one of the oldest approved chemotherapeutic agents, are used in about 50% of chemotherapies administered to cancer patients (2). Nevertheless, treatment with cisplatin is associated with significant problems. As a DNA-damaging agent, cisplatin kills not only cancer cells but also healthy cells. Off-target effects can be irreversible and include hearing loss and neuropathy (3). Moreover, while cisplatin treatment has been shown to be effective in reducing the mortality of testicular, bladder, cervical, and head and neck cancer, other tumors such as lung cancer are innately resistant toward cisplatin (4, 5). Finally, many cancer types have been reported to develop cisplatin resistance coinciding with long term treatment. For example, ovarian cancer cells may respond well during initial chemotherapy treatment but commonly become resistant when the cancer reoccurs (5). Thus, the non-specific toxicity of cisplatin and the resistance of some cancers restrict the therapeutic use of the drug. To increase the treatment options for cancer patients, it is therefore of crucial importance to understand how mammalian cells respond to cisplatin-mediated toxicity, and hence identify targets that might increase cisplatin sensitivity specifically in cancer cells.

The toxicity of cisplatin is attributed primarily to its interaction with DNA, and the cisplatin-induced formation of DNA adducts and double strand breaks (6, 7). This DNA damage, if not properly mended by the cellular DNA repair machinery, blocks cell cycle progression and induces apoptosis (8). However, induction of apoptosis has also been observed in cells lacking a nucleus (9), implying that cisplatin might target other cellular components in addition to nuclear DNA. Based on recent work which showed that cisplatin also interacts with mitochondrial DNA, proteins, and small molecules such as glutathione (10–13), it was proposed that mitochondrial dysfunction and/or oxidative stress contribute to cisplatin toxicity. These unexpected results emphasize the need to further elucidate the mechanism of cisplatin action.

Our lab has recently discovered that bacteria respond to oxidative stress treatment with the accumulation of large amounts of polyP, linear chains of phosphoanhydride bond-linked phosphates, which serve to protect proteins and cells against irreversible oxidative damage (14). Mammalian cells also synthesize polyP, but functional studies focused primarily on its roles in cell proliferation, apoptosis, angiogenesis, metastasis, blood clotting, and inflammation (15–21). Here we set out to test whether polyP plays a role during cisplatin-induced stress in mammalian cells. Our studies revealed that several cancer cell lines respond to cisplatin treatment with a robust upregulation and cellular redistribution of endogenous polyP, leading to the formation of distinct nucleolar polyP foci. Instead of the expected cytoprotective function of polyP, however, we discovered that the levels of endogenous polyP directly correlate with the levels of caspase-mediated apoptosis induction, suggesting that polyP plays a role in cisplatin-mediated apoptosis. Indeed, by administrating exogenous polyP, we were able to increase cisplatin-induced toxicity, suggesting that altering cellular polyP levels might represent a potential novel mechanism to modulate the therapeutic efficacy of cisplatin.

## Materials and Methods

### Reagents, Plasmids, and Proteins

Polyphosphates with chain lengths of 14, 60, 130, and 300 P_i_ were kindly provided by T. Shiba (RegeneTiss, Japan) and aliquoted to avoid repeated freeze-thaw cycles. A cis-Diammineplatinum(II) dichloride (Sigma-Aldrich) stock solution (3 mM) was prepared in double distilled water. The plasmid pETM41-*Ec*PPXc, which encodes the Maltose Binding Protein (MBP)-*Ec*PPXc was a kind gift from Florian Freimoser (Addgene plasmid #38329; http://n2t.net/addgene:38329; RRID:Addgene_38329) (22). To generate the mCherry-*Ec*PPXc fusion protein, *Ec*PPXc was cloned into the mCherry-containing pTEV5 vector between BamHI and NotI sites. To generate the alternative GFP-*Ec*PPXc probe, GFP was amplified from the pEGFP-N2 vector with flanking regions containing NdeI and BamHI recognition sequences, and inserted to replace mCherry in the mCherry-*Ec*PPXc construct. The purification of His-tagged mCherry-*Ec*PPXc, GFP-*Ec*PPXc, and their respective His-tagged control protein mCherry and GFP was done using a Ni-NTA column (Qiagen, Hilden, Germany). All reagents were purchased from Thermo Fisher Scientific (Waltham, MA, USA), Sigma-Aldrich (St. Louis, MO, USA) or New England Biolabs (Ipswich, MA, USA) unless specified otherwise.

### Cell Lines

HeLa EM2-11ht cell line was a generous gift of Dr. J. Nandakumar, University of Michigan (23). HeLa EM2-11ht and HeLa (ATCC® CCL-2™, ATTC, Manassas, VA, USA) cells were grown in DMEM (#11995065, Thermo Fisher Scientific). The ovarian cancer cell lines OVCAR3 and OVCAR8 (a generous gift of Dr. K. McLean, University of Michigan) were cultured in DMEM (ATCC® 30-2002™) from ATTC (Manassas, VA, USA). All media were supplemented with 10% Fetal Bovine Serum (#F4135, Sigma-Aldrich) and 1% Penicillin-Streptomycin (#15140122, Thermo Fisher Scientific). Cells were grown at 37°C with 5% CO_2_.

### Cisplatin Treatment

Cells grown to ~80% confluence were detached from the flask using 0.25% Trypsin-EDTA (#25200056, Thermo Fisher Scientific). 1.2 × 10^5^ cells were seeded in each well of a 24-well plate and 10^4^ cells were plated in each well of a 96-well plate. After the cells were allowed to attach overnight, they were treated with a range of cisplatin concentrations for the indicated period of time.

### Proliferation and Cell Death Assays

All microplate reader-based assays were performed in 96-well cell culture plates (Corning, NY, USA). The WST-1 Cell Proliferation Assay Kit (#10008883, Cayman Chemical, Ann Arbor, Michigan, USA) was used to assess proliferation. Briefly, medium containing cisplatin was removed from the 96-well plate, and the mixture of the WST-1 Developer Reagent and the Electron Mediator Solution was first diluted in fresh medium to the working concentration and then added to the treated cells. Absorbance was measured in a BMG FLUOstar Omega microplate reader (Ortenberg, Germany) at a wavelength of 450 nm. Using the average of three technical replicates, the absorbance readings of the treated samples were normalized to those of the untreated cells.

To determine the fraction of cells with compromised cell membranes as a readout for cell death, the SYTOX™ Green Nucleic Acid Stain (#S7020, Thermo Fisher Scientific) was used (24). All cisplatin treatments were set up in duplicates in a 96-well plate. Following the treatment, one replicate was permeabilized with 120 μM Digitonin (#D141, Sigma-Aldrich) while the other remained unperturbed. Then, 5 μM SYTOX™ Green Nucleic Acid Stain was added to all wells, and the samples were incubated for 30 min at 37°C. The fluorescence of the incorporated SYTOX™ Green Nucleic Acid Stain was measured in a Tecan Infinite M1000 microplate reader (Männedorf, Switzerland) using an excitation wavelength of 504 nm and an emission wavelength of 523 nm. With the fluorescence from the digitonin-treated samples serving as the readout for total number of cells, the percentage of dead cells was calculated by normalizing the fluorescence of the unperturbed wells to the fluorescence of the corresponding digitonin-treated wells.

The percentage of dead cells was also determined using Trypan Blue, which stains cells with disrupted membranes. Treated cells were detached from the wells using 0.25% Trypsin-EDTA, and an aliquot of cells was mixed and incubated shortly with Trypan Blue Solution (#15250061, Thermo Fisher Scientific). Stained (i.e., dead) cells and non-stained (i.e., live) cells were counted in a light microscope and the percentage of dead cells was calculated.

To determine the extent of apoptosis induction, a luminescence-based activity assay for the executioner caspases 3 and 7 was utilized. Cells were seeded at a density of 10^4^ cells per well in a 96-well white clear bottom plate (Greiner Bio-One, Monroe, NC, USA). The Caspase-Glo® 3/7 assay (#G8090, Promega Corporation, Madison, WI, USA) was used to measure the activity of caspase 3 and caspase 7. The manufacturer's instructions were followed with the exception that only 70 μl of the Caspase-Glo 3/7 Reagent was added to 70 μl of fresh medium to each well. After incubating the plate for 30 min at room temperature, luminescence was measured at 23°C in a BMG FLUOstar Omega microplate reader (Ortenberg, Germany). Each treatment was performed with three technical replicates and the average was calculated.

### Flow Cytometry

Following cisplatin treatment, cells were detached using 0.25% Trypsin-EDTA and combined with the supernatant, which contained dead, floating cells. Then, all cells were spun down at 300 g at room temperature for 5 min, and the pellet was washed with Cell Staining Buffer (#420201, BioLegend, San Diego, CA, USA). For the labeling of apoptotic and necrotic cells, the FITC Annexin V Apoptosis Detection Kit with PI (#640914, BioLegend, San Diego, CA, USA) was used, following the manufacturer's protocol. In short, the cell pellet was resuspended in Annexin V Binding Buffer. Then, Annexin V and Propidium iodide (PI) were added to the cell suspension. The percentage of Annexin V- and PI-positive cells (i.e., late apoptotic or necrotic cells) was measured in an Attune NxT Flow Cytometer (Thermo Fisher Scientific, Waltham, MA, USA; courtesy of the Buttita Lab, University of Michigan, Ann Arbor, MI, USA). Annexin V was measured in the BL1 channel while PI was measured in the BL3 channel. Gating was done using Annexin V-single stained cells, PI-single stained cells, and a double positive control.

### Immunofluorescence Staining

For fluorescence microscopy, cells were seeded onto 12 mm cover slips (#CLS-1760-012, Chemglass Life Sciences, Inc., Vineland, NJ, USA) placed in a 24-well plate (Corning, NY, USA). Following cisplatin treatment, cells were fixed in 4% v/v freshly prepared paraformaldehyde (#1578100, Electron Microscopy Sciences, Hatfield, PA, USA) at room temperature for 15 min, washed with PBS, and permeabilized in 0.3% Triton X-100 (#0219485480, MP Biomedicals, Solon, OH, USA) for 10 min. Triton X-100 was prepared in blocking solution, which contained 1% bovine serum albumin (#A3059, Sigma-Aldrich) in PBS. After a brief wash, cells were incubated in blocking solution for 1 h at room temperature. For the visualization of endogenous polyP, mCherry-*Ec*PPXc, or GFP-*Ec*PPXc, and the respective control protein mCherry or GFP, were used at a concentration of 10 μg/ ml in blocking solution and incubated overnight at 4°C protected from light. For the staining of apoptotic cells, a rabbit cleaved caspase-3 (Asp175) monoclonal antibody (#MAB835, R&D Systems, Minneapolis, MN, USA) was used at a concentration of 8 μg/ ml in blocking solution. For the staining of p53, a mouse p53 antibody (#DO-1, Santa Cruz Biotechnology, Dallas, TX, USA) was used at a concentration of 4 μg/ ml. To stain RNA polymerase I (RNA pol I), a rabbit anti-PAF49 IHC antibody (#IHC-00474, Bethyl Laboratories, Montgomery, TX, USA) was used at a concentration of 8 μg/ ml. To label nucleophosmin (NPM1), an anti-NPM1 antibody (#32-5200, Thermo Fisher Scientific) was used at a concentration of 2 μg/ ml. Incubation of these primary antibodies was performed overnight at 4 °C together with polyP staining. The next day, cells were washed with PBS and then incubated with the respective secondary antibody for 1 h at room temperature protected from light. Secondary antibodies used in this study were obtained from Abcam, Cambridge, United Kingdom, and included goat anti-rabbit IgG-Alexa Fluor® 488 (#ab150077), donkey anti-mouse IgG-Alexa Fluor® 488 (#ab150105), and goat anti-rabbit IgG-Alexa Fluor® 647 (#ab150079). After washing with PBS, cells were incubated with DAPI (#D1306, Thermo Fisher Scientific) at a concentration of 2.5 μg/ ml for 10 min at room temperature to stain the nucleus. Cells were washed five times with PBS before the coverslips were mounted on microscope objective slides using Citiflour AF1 mounting medium (#19470, Ted Pella, Inc., Redding, CA, USA).

Fluorescence images for mCherry-*Ec*PPXc staining were acquired with a 40× objective on an Olympus BX61 (Olympus, Center Valley, PA, USA) upright microscope equipped with a Photometrics Coolsnap HQ2 cooled CCD camera and a quad filter set (DAPI/ FITC/ TRITC/ CY-5). A closed feedback loop was enabled to keep the illumination of the X-Cite® exacte mercury lamp consistent. GFP-*Ec*PPXc labeling and co-localization analysis of polyP, RNA polymerase I and NPM1 were performed with a Leica SP8 laser scanning confocal microscope (Leica GmbH, Mannheim Germany) on a DMI8 microscope base using LAS X software, a 100× oil objective (Leica 11506378) and a 405 nm diode laser, in addition to a multi-line white light laser, set to 488, 594, and 647 nm excitation wavelengths. Spectral detection using a PMT from 410 to 480 nm was utilized for DAPI, a HyD detector from 493 to 560 nm for GFP and Alexa Fluor® 488, and a HyD detector from 653 to 800 nm for Alexa Fluor® 647.

### Uptake of Fluorescently-Labeled PolyP

PolyP_300_ was end-labeled with Alexa Fluor™ 647 Cadaverine (#A30679, Thermo Fisher Scientific, Waltham, MA, USA) as described in (25). A labeling efficiency of 10–15% was obtained. HeLa cells were seeded on coverslips as described above. After washing the cells with PBS, they were incubated with 200 μM labeled polyP (in Pi units) overnight in the absence or presence of 25 μM cisplatin. Staining for endogenous polyP was performed as described above using the GFP-*Ec*PPXc probe.

### Image Quantification

**Figure 1:**
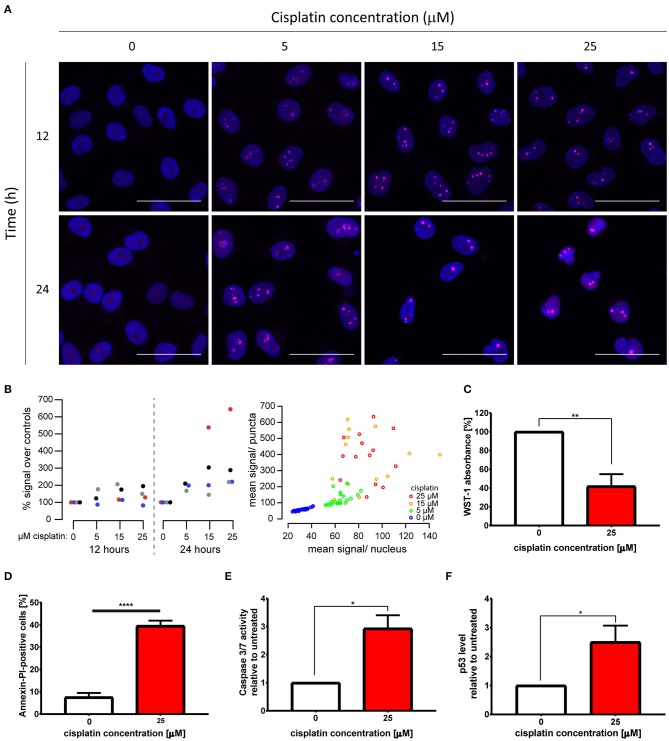
Cytotoxic cisplatin treatment triggers formation of nuclear polyP foci. **(A)** Visualization of endogenous polyP levels and distribution in HeLa cells treated with 0, 5, 15, and 25 μM cisplatin for 12 and 24 h, respectively. The overlay of GFP-*Ec*PPXc channel (magenta) and DAPI signal (blue) reveals the nuclear localization of polyP. Representative images of projected z series are displayed. Scale bar: 50 μm. **(B)** Quantification of polyP levels following the same cisplatin treatments as those described in **(A)**. Left: Four replicate experiments are shown, color coded per experiment. Mean GFP-*Ec*PPXc signal increased as [cisplatin] increased: *p* = 0.0367 at 24 h by repeated measures ANOVA. Measurements at 12 h were not significantly different. Right: From one representative experiment, the mean GFP-*Ec*PPXc signal of puncta regions is plotted against the mean value of the non-puncta regions of each nucleus. **(C–F)** Evaluations of cell viability after 25 μM cisplatin treatment for 24 h (red bars). **(C)** Proliferation was determined by WST-1 assay (*N* = 6, mean + s.e.m., unpaired *t*-test, **p = 0.0011). **(D)** Percentage of Annexin V and PI-positive cells was measured by flow cytometry (*N* = 4, mean + s.e.m., unpaired *t*-test, ****p < 0.0001). Apoptosis induction was monitored by **(E)** activity of caspases 3 and 7 (*N* = 3, mean + s.e.m., unpaired *t*-test, *p = 0.0147), and **(F)** p53 level (*N* = 6, mean + s.e.m., unpaired *t*-test, *p = 0.0241).

Immunofluorescence images of DAPI and mCherry-*Ec*PPXc (or GFP-*Ec*PPXc) were used to define the puncta regions and quantify their signal using Fiji (ImageJ) software. Briefly, nuclear regions of interest (ROIs) were defined in the DAPI channel, using *Thresholded Blur, Make Binary*, and *Fill Holes* followed by hand segmentation to ensure accurate ROIs, and applied to the mCherry-*Ec*PPXc (or GFP-*Ec*PPXc) channel. Total nuclear signal was measured. Then the nuclear ROIs were separated into distinct files, and each nuclear ROI was used to define puncta ROIs. Puncta ROIs were found by applying several ImageJ plugins in order: *Median* and *Thresholded Blur* to reduce background noise and maintain puncta edges, *Subtract Background* with a rolling ball radius of 5 pixels to define areas that are brighter than nucleoplasm signal, and finally *Make Binary, Fill Holes*, and *Watershed* to define the puncta ROIs. These puncta ROIs were then applied to the original mCherry-*Ec*PPXc (or GFP-*Ec*PPXc) channel to record size and pixel intensity information. Finally, nucleoplasm signal was measured by defining a nucleoplasm ROI: Nuclear ROI with holes for all the puncta ROIs found in that nucleus. This was measured as for the puncta ROIs. Image background signal was determined by defining five non-cellular ROIs per image, and averaging the pixel intensity. This background was subtracted from all ROI data. R Studio was used to analyze puncta data and produce the dot plot in Figure 1 and the histograms and dot plot in Supplementary Figure 1. (https://www.rstudio.com).

### Generation of Stable *Sc*PPX-Expressing Cells

*Sc*PPX, with an N terminal Nuclear Localization *S*equence (NLS) followed by three FLAG tags, was cloned into pBI-4 vector using NotI and SalI, and subsequently inserted into pBI-F3 vector using SarI and StuI. HeLa EM2-11ht cells were seeded in a 6-well plate with the density of 3 × 10^5^ cells/ well and transfected the next day with 1 μg pCAGGS-IRES-Puro plasmid and 1 μg NLS-3× FLAG-*Sc*PPX plasmid using Lipofectamine™ 3000 (#L3000008, Thermo Fisher Scientific) following the manufacturer's protocol. After 12 h of incubation with the transfection mix, cells were transferred to a 10 mm culture dish and grown in the presence of 5 μg/ ml puromycin dihydrochloride (#P8833, Sigma-Aldrich). After 36 h, puromycin was removed, cells were rinsed with PBS, and then grown in medium supplemented with 40 μM ganciclovir (#G2536, Sigma-Aldrich) for 8–10 days until single colonies appeared. Single colonies were picked, and grown in the absence or presence of 200 ng/ ml doxycycline hyclate (#D9891, Sigma-Aldrich) to induce *Sc*PPX protein expression. The expression of NLS-3× FLAG-*Sc*PPX was verified with Western Blotting using a mouse anti-FLAG M2 antibody (#F3165, Sigma-Aldrich). All vectors were kind gifts of Dr. J. Nandakumar, University of Michigan.

### *Ex vivo Sc*PPX Activity Assay

NLS-3× FLAG-*Sc*PPX transgenic HeLa EM2-11ht cells were seeded in a 6-well plate at the density of 1.5 × 10^5^ cells/ well. The next day, expression of *Sc*PPX was induced with 200 ng/ ml doxycycline for 48 h, after which 25 μM cisplatin was added and cells were incubated for another 24 h. Cells were detached using 0.25% Trypsin-EDTA, collected with PBS and spun down at 300 g at room temperature for 5 min. The pellet was washed once with 100 μl lysis buffer: 20 mM Tris-HCl pH 7.5, 50 mM NaCl, 2 mM EDTA, 1% IGEPAL® CA-630 (#I8896, Sigma-Aldrich), Complete™, EDTA-free Protease Inhibitor Cocktail (#5056489001, Sigma-Aldrich), 12.5 units/ ml Pierce™ Universal Nuclease for Cell Lysis (#88702, Thermo Scientific) and spun down at 300 g at 4°C for 5 min. The pellets were resuspended in 100 μl lysis buffer and cells were lysed by sonication at 4°C. The activity of *Sc*PPX was measured using the EnzChek® Phosphate assay kit (#E6646, Thermo Fisher Scientific) following the manufacturer's instructions. As substrate for *Sc*PPX, 150 μM polyP_130_ was added to the reaction mix, and the release of phosphate was monitored by absorbance reading in a Synergy HTX multi-mode microplate reader (BioTek, Winooski, VT, USA). For the normalization of input, the protein amount of each sample was measured using the *DC*™ protein assay (#5000112, Bio-Rad Laboratories, Hercules, CA, USA) following the manufacturer's instructions, and absorbance was determined at 700 nm in a BMG FLUOstar Omega microplate reader (Ortenberg, Germany). The specific activity of *Sc*PPX was calculated from the initial rate of polyP_130_ degradation and the protein concentration of the cell lysate.

### Statistics

If not noted otherwise, the average of N > 3 biological replicates and the s.e.m. are plotted. Graphing and statistics were performed using GraphPad Prism 7 (San Diego, CA, USA). Number of replicates and significance level are found in each figure legend.

## Results

### Cisplatin Treatment Triggers PolyP Accumulation in Cancer Cells

To study the effects of cisplatin treatment on the levels and distribution of polyP in cancer cells, we treated the cervical cancer cell line HeLa EM2-11ht (from here on referred to as HeLa) with 0, 5, 15, and 25 μM cisplatin for 12 and 24 h, and visualized endogenous polyP using the specific probe GFP-*Ec*PPXc. This fusion protein consists of the C-terminal polyP-binding domain of *Escherichia coli* exopolyphosphatase (*Ec*PPXc) (22, 26) and the fluorescent protein GFP. Staining of fixed cells with GFP lacking the *Ec*PPXc domain revealed a negligible background signal in both untreated and cisplatin-treated cells (Figure S1A). In contrast, however, staining with the polyP-specific probe GFP-*Ec*PPXc revealed polyP signals in the nucleus of unstressed HeLa cells (Figure 1A). These signals dramatically increased upon cisplatin treatment in a dose- and time-dependent manner and presented themselves as distinct and bright nuclear foci (Figure 1A). The apparent cisplatin-induced accumulation of endogenous polyP was significant and consistent across experiments (Figure 1B) as well as in another HeLa clone obtained from the American Type Culture Collection (Figure S1B). In-depth quantification of the distribution and intensity of nuclear polyP puncta of four experiments revealed that following cisplatin stress the number of polyP puncta per cell drastically decreased while the size of the puncta and their fluorescence intensity significantly increased (Figure S1C). These results indicate that the cellular polyP pool not only increases but significantly rearranges its subcellular localization upon cisplatin exposure.

To characterize the viability of the cells following the treatment, we focused on treatment conditions which led to the most profound changes in cellular polyP in HeLa cells, that is, 25 μM cisplatin treatment for 24 h. We observed that this treatment was sufficient to significantly impair the proliferation of HeLa cells, causing a 60% reduction in cell numbers compared to the untreated control (Figure 1C). Cisplatin-treated cells also displayed, on average, a 5-fold induction of cell death as measured by the percentage of Annexin V and Propidium Iodide-positive cells (Figure 1D) as well as Trypan Blue-positive cells (Figure S1D). Moreover, treatment with cisplatin caused a 3-fold increase in both caspase 3 and 7 activity (Figure 1E), doubled the amount of cleaved caspase-3 (Figure S1E), and elevated the levels of the tumor suppressor p53 when compared to the untreated control (Figure 1F). Based on these results, we concluded that the cisplatin treatment we used to trigger a robust polyP response concomitantly inhibits proliferation and induces cell death through caspase activation and p53 stabilization in HeLa cells.

### Cisplatin Treatment Leads to Nucleolar PolyP Foci Formation

**Figure 2:**
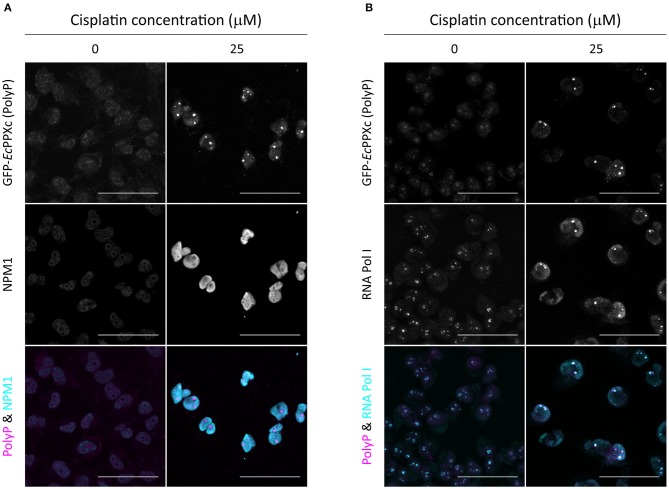
Cisplatin-induced polyP foci are adjacent to RNA Pol I in the nucleolus. Co-staining of untreated and cisplatin-treated HeLa cells with GFP-*Ec*PPXc and **(A)** an NPM1 antibody or **(B)** an RNA Pol I antibody. The overlay of polyP (magenta) and the respective nucleolar marker (cyan) is shown to illustrate the localization of polyP foci in the nucleolus. Representative images of projected z series are displayed. Scale bar: 50 μm. The corresponding control images labeled with GFP and the fluorescent secondary antibodies are shown in Figures S2A,B.

By using the nuclear DNA stain DAPI as reference, we found that cisplatin-induced polyP foci mainly localize to the nucleoli of HeLa cells (Figure 1A). To further investigate the subcellular localization of polyP following cisplatin treatment, we co-stained control and cisplatin-treated HeLa cells with GFP-*Ec*PPXc and an antibody against nucleophosmin (NPM1), a protein localized to the granular component of the nucleoli (27). In untreated HeLa cells, we did not observe any significant overlap of GFP-*Ec*PPXc fluorescence with NPM1 antibody signal (Figure 2A, left panel, and Figure S2A for controls). Upon cisplatin exposure, NPM1 re-localizes within the nucleus, like polyP. However, in contrast to polyP, NPM1 diffuses into the nucleoplasm and hence does not co-localize with polyP (Figure 2A, right panel). These results excluded the possibility that polyP is enriched in the granular component of the nucleoli. To test whether polyP localizes to the fibrillar center or dense fibrillar component, sub-compartments of the nucleolus in which rDNA transcription and early processing take place (27), we conducted similar co-staining experiments but used an antibody against RNA polymerase I, a component of the fibrillary center of nucleoli instead (28). As shown in Figure 2B (and Figure S2B for controls), we found a significant, albeit partial overlap between polyP (magenta) and RNA Pol I (cyan) fluorescence upon cisplatin treatment. These results were consistent with previous studies which showed co-localization of polyP and RNA Pol I in myeloma cells (29). Interestingly, the authors also observed a direct inhibition of RNA Pol I activity by polyP *in vitro*. Given that cisplatin is known to halt rRNA transcription in the concentration range of 25 to 100 μM *in vivo* (30), our finding that polyP and RNA Pol I are in close proximity after cisplatin treatment suggests that polyP and RNA Pol I might be physically interacting and/or functionally related. Yet, more vigorous biochemical analyses are needed to test this hypothesis.

### Cisplatin-Induced PolyP Responses Positively Correlate With Cellular Toxicity

**Figure 3:**
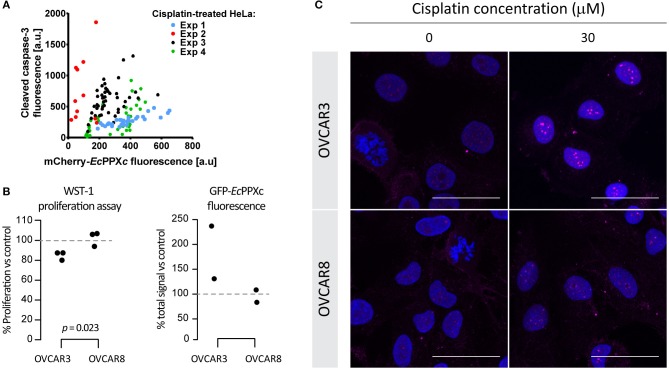
Correlation between cisplatin-induced polyP accumulation and cellular toxicity. **(A)** Correlation of polyP levels and cleaved caspase-3 levels in individual HeLa cells treated with 25 μM cisplatin for 24 h. Four different experiments were conducted and symbols of the same color represent cells from the same experiment. **(B)** A positive correlation between nuclear polyP levels and cisplatin-triggered proliferation inhibition in ovarian cancer cells. Cisplatin-sensitive cell line OVCAR3 and cisplatin-resistant cell line OVCAR8 were treated with 30 μM cisplatin for 8 h. Cisplatin toxicity was indicated by reduced viability of cisplatin-treated cells with the WST-1 assay (left panel). *P*-values derived from two-tailed *t*-tests. And average nuclear polyP levels, normalized to untreated controls, were measured by GFP-*Ec*PPXc fluorescence intensity (right panel). **(C)** Visualization of endogenous polyP in ovarian cancer cells with varying degrees of cisplatin sensitivity. OVCAR3 and OVCAR8 cells were subject to the same treatment as that in **(B)**. Images from a representative experiment are shown to demonstrate the distinct polyP responses of OVCAR3 and OVCAR8 cells in response to cisplatin, revealed by the different intensities and distribution of GFP-*Ec*PPXc signals (magenta). Nuclear DNA was labeled with DAPI. Representative images of projected z series are displayed. Scale bar: 50 μm.

Microscopic analysis of cisplatin-treated cells revealed a significant cell-to-cell variation in polyP staining after treatment with 25 μM cisplatin for 24 h. Intriguingly, we also observed significant cell-to-cell variation in the cleaved caspase-3 signal, suggesting a potential correlation between polyP levels and cleaved caspase-3 signal (and hence initiation of apoptosis). To investigate whether a correlation between polyP levels and cleaved caspase-3 levels exists, we determined the amount of cleaved caspase-3 signal and that of mCherry-*Ec*PPXc signal after cisplatin treatment in four independent experiments using between 12 and 46 individual cells per experiment. These measurements revealed that, on average, cells with higher polyP levels have increased levels of cleaved caspase-3 (Figure 3A). It is of note that cisplatin-induced cleaved caspase-3 and polyP do not co-localize to the same cellular compartment after 24 h of cisplatin exposure (Figure S2C), rendering a direct interaction between the two molecules very unlikely.

The observed positive correlation between cisplatin-mediated polyP accumulation and apoptosis initiation in HeLa cells prompted us to explore other cancer cells with intrinsically different sensitivities to cisplatin, and compare their polyP responses after the drug treatment. Based on IC_50_ values reported in the literature, ovarian cancer cell line OVCAR3 is 1.4 to 2-fold more susceptible to cisplatin than the ovarian cancer cell line OVCAR8 (31, 32). We confirmed these results with WST-1 proliferation assays, which showed that OVCAR8 cells are indeed more resistant to cisplatin treatment compared to OVCAR3 cells (Figure 3B, left panel). Similar to HeLa cells, both ovarian cancer cell lines responded to cisplatin treatment with a dose- and time-dependent increase in nuclear polyP levels as well as the formation of distinct nucleolar polyP foci (Figures S3A,B). However, and in agreement with our previous results, the extent to which OVCAR8 cells responded to cisplatin treatment with the upregulation and redistribution of polyP was much less pronounced compared to OVCAR3 cells (Figure 3B, right panel and Figure 3C). In fact, at least twice the amount of cisplatin was necessary to trigger a polyP-response in OVCAR8 cells that was comparable to what we observed in OVCAR3 cells (Figures S3A,B). These results supported our previous conclusion that cisplatin-mediated polyP accumulation and rearrangement positively correlates with the level of apoptosis induction and hence cisplatin sensitivity. Although the underlying mechanism remains elusive, the dynamic changes of nuclear polyP appear to be a *bona fide* response to cisplatin-induced toxicity in various carcinomas and related to the susceptibilities of cancer cells to the chemotherapeutic agent.

### Exogenous PolyP Administration Increases Cisplatin Sensitivity of Select Cancer Cells

Based on our observation that the accumulation of endogenous polyP correlates with the induction of apoptosis upon cisplatin exposure (Figure 3), we investigated whether manipulating cellular polyP levels would alter cisplatin sensitivity. Unfortunately, the genetic manipulation of *in vivo* polyP levels in mammalian systems is hampered by the fact that the polyP-producing and -decomposing enzyme(s) are still unknown (33). In an attempt to decrease endogenous polyP levels, we expressed the *Saccharomyces cerevisiae* exopolyphosphatase (*Sc*PPX) under a doxycycline-inducible promoter (23) specifically in the nucleus of HeLa cells. Similar strategies to decrease cellular polyP have been used previously albeit with variable success (16, 34, 35). We confirmed the doxycycline-induced expression of *Sc*PPX in the stable cell clones (Figure S4A), and subsequently measured the enzymatic activity of *Sc*PPX in cell lysates by monitoring the degradation of chemically synthesized polyP. Comparing *Sc*PPX specific activity in cell lysates of untreated cells (370 mmol Pi·min^−1^·g^−1^) vs. cisplatin-treated cells (449 mmol Pi·min^−1^·g^−1^) revealed that the yeast enzyme was fully functional in the presence of cisplatin. However, we failed to observe a >10% reduction in the amount of polyP that accumulated in the *Sc*PPX-expressing cells following cisplatin treatment (Figure S4B). As a result, there was no significant difference in the cisplatin-induced proliferation deficiency between the *Sc*PPX-expressing cells and their parent cells either (Figure S4C).

**Figure 4:**
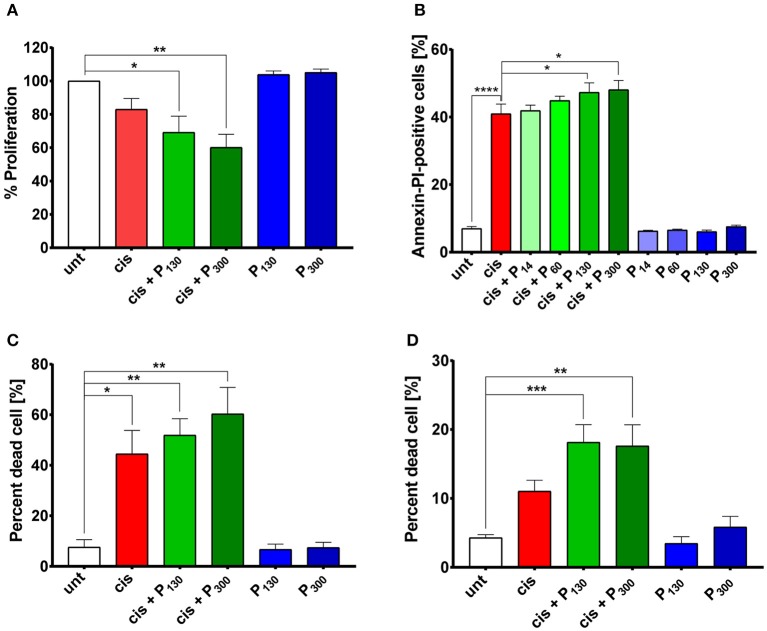
Exogenous polyP increases cisplatin-induced cellular toxicity. **(A)** Proliferation of HeLa cells treated with 5 μM cisplatin in the absence (red bars) or presence of polyP (green bars) or exposed to 200 μM polyP_130_ or polyP_300_ only (blue bars) (*N* = 5, mean + s.e.m). A one-way ANOVA followed by a Tukey multiple comparison test was performed (*p < 0.05; **p < 0.01). **(B)** Percentage of Annexin V and PI-positive HeLa cells following the combined treatment of 25 μM cisplatin and 200 μM exogenous polyP_14_-_300_ (shades of green) compared to cisplatin only treatment (red bar) and polyP alone control (blue bars) (*N* = 7, mean + s.e.m., one-way ANOVA with Dunnett's multiple comparison, *p < 0.05; ****p < 0.0001). **(C)** Percentage of SYTOX Green-permeable (i.e., dead) HeLa cells simultaneously treated with 25 μM cisplatin and 200 μM polyP_130_ or polyP_300_ (green bars). Treatment with 25 μM cisplatin alone is shown in red and the polyP only control is shown in blue (*N* = 3; mean + s.e.m.). A one-way ANOVA followed by a Tukey multiple comparison test was performed (*p < 0.05; **p < 0.01). **(D)** Percentage of SYTOX Green-permeable (i.e., dead) ovarian cancer cell line OVCAR3 simultaneously treated with 25 μM cisplatin and 200 μM polyP_130_ or polyP_300_ (green bars), or exposed to either 25 μM cisplatin (red bar) or polyP only (blue bars) (*N* = 3; mean + s.e.m.). A one-way ANOVA followed by a Tukey multiple comparison test was performed (**p < 0.01; ***p < 0.001).

Being unable to significantly decrease endogenous polyP levels, we employed the opposite approach and determined whether increasing endogenous polyP levels might alter the cisplatin sensitivity of HeLa cells. We and others have previously shown that mammalian cells readily take up exogenous polyP (Figure S4D) (36). We therefore exposed HeLa cells simultaneously to cisplatin and exogenous polyP, and determined whether polyP supplementation affects the sensitivity of cells toward cisplatin. Indeed, HeLa cells treated with 5 μM cisplatin in combination with either 200 μM polyP_130_ or polyP_300_ showed a more severe growth defect compared to those treated with cisplatin alone (Figure 4A). Cytotoxicity assays confirmed these results and showed that the presence of physiologically relevant chain lengths of polyP, that is, polyP_130_ and polyP_300_ significantly increased the efficacy of a higher cisplatin dose, i.e., 25 μM, in triggering apoptosis and eventually cell death (Figures 4B,C). Notably, we did not observe any effect when we supplemented cisplatin treatment with shorter polyP chains (polyP_14_ and polyP_60_), a finding that was fully consistent with previous reports that physiological chain lengths of polyP (>60 Pi units) are disproportionally more effective than short polyP chains in a variety of polyP functional assays (25). Moreover, we excluded that incubation of cells with polyP in the absence of cisplatin affects cell growth or survival (Figures 4A–C). To test whether polyP enhances cisplatin efficacy also in other cell lines, we co-treated OVCAR3 cells with both polyP and cisplatin (Figure 4D). Again, we observed a significantly increased toxicity of cisplatin when the treatment was combined with polyP. These results strongly suggest that non-toxic, physiologically relevant polyP levels and chain lengths are able to synergistically increase the cytotoxicity of cisplatin in cancer cells.

## Discussion

In this study we discovered that several different cancer cell lines respond to cisplatin treatment with the accumulation of endogenous polyP, whose relative cellular levels appeared to directly correlate with apoptosis induction and cell death. Cisplatin treatment seemed to trigger both new polyP synthesis as well as subcellular reorganization of polyP pools into distinct nucleolar foci, coinciding with a general cisplatin-induced reorganization of the nucleoli. These membraneless compartments, which are the birthplace of the ribosomes, have previously been shown to be sensitive to perturbations in metabolic rates, cellular stress, and DNA damage (27, 37). Here, we report the identification of cisplatin-induced polyP foci primarily in the fibrillar center and dense fibrillar component, the regions of rDNA transcription and early processing (27). These results suggested that polyP might be involved in the process and/or regulation of ribosomal RNA synthesis, a conclusion that was further supported by our findings that polyP partially co-localizes with RNA Pol I upon cisplatin stress. Intriguingly, previous unrelated studies showed (i) that polyP directly inhibits RNA Pol I activity *in vitro* (29), and (ii) that cisplatin treatment inhibits the activity of RNA Pol I *in vivo* (38). Based on our results and the finding that cisplatin-induced polyP levels and apoptosis induction are directly linked, it is now tempting to speculate that cisplatin-induced polyP accumulation affects the activity of RNA Pol I, thereby activating the apoptotic program. It is of note that reduction of RNA Pol I-mediated transcription triggers p53-mediated apoptosis in lymphoma cells but not in non-tumor cells (39). This specificity raises the intriguing possibility that polyP might affect RNA Pol I activity and enhance cisplatin toxicity specifically in cancer cells. In support of this notion, a recent report found that the administration of polyP triggered apoptosis in human colon cancer but failed to induce apoptosis in primary cultures of murine small intestinal epithelial cells (40).

In support of our hypothesis that polyP plays a role in inducing and/or regulating the progression of apoptosis following cisplatin stress, we found that cells with higher levels of endogenous polyP had, on average, higher levels of apoptosis (Figure 3). Cleaved caspase-3 and cisplatin-induced polyP puncta, however, do not localize to the same compartment (Figure S2C), raising the question whether polyP acts on other steps in this pathway. The nucleolus has long been implicated to partake in the execution of apoptosis (41). Intriguingly, many nucleolar proteins, including Topoisomerase I (Top1), a potential substrate of caspase-3 (42, 43), have recently been found to be polyphosphorylated, a novel post-translational modification characterized by the addition of polyP chains to lysines (44). Functioning as a molecular switch, polyphosphorylation not only appears to mediate the translocation of substrates in and out of the nucleolus but also governs their functional activities. Hence it will be of interest to determine whether accumulation of polyphosphorylated components lead to the observed puncta formation and contribute to the mechanism by which polyP modulates the course of apoptosis.

Our observation that cleaved caspase-3 and endogenous polyP levels positively correlate now raises the fundamental question whether cells that innately generate more polyP are more sensitive to cisplatin (i.e., polyP being a mediator of apoptosis), or whether cells that are more sensitive to cisplatin accumulate more polyP (i.e., polyP being a “by-product” of apoptosis induction). A key experiment that would help to answer this question is to test the cisplatin sensitivity of polyP-depleted cells. However, the process of how polyP is generated in mammalian cells has yet to be discovered (33). Unable to manipulate the mammalian players of polyP synthesis, we therefore attempted to modulate endogenous polyP by expressing a polyP-degrading enzyme from yeast (16). This approach, however, was unsuccessful in reducing endogenous polyP levels or affecting cisplatin sensitivity in our study. Since the inability to reduce cellular polyP levels was not due to potential inhibitory effects of cisplatin on the enzymatic activity of *Sc*PPX, we now speculate that cisplatin-induced polyP might either not be accessible to exopolyphosphatases *in vivo* (45), or that the spatial confinement of polyP in the interior of the nucleolus might protect polyP from *Sc*PPX (35). Indeed, when we probed the subcellular localization of polyP and *Sc*PPX, we found that the two molecules did not partition to the same region in the nucleus. While endogenous polyP was highly enriched in the nucleolus, *Sc*PPX was most abundant in the cytosol and nucleoplasm (Figure S4E). Furthermore, we cannot rule out the possibility that the polyP-degrading enzyme, *Sc*PPX, and the polyP-binding probe, *Ec*PPXc, recognize different fractions of polyP in the cells. The former might be capable of scavenging soluble polyP (exemplified by polyP in the mitochondrial matrix) (46), whereas the latter might selectively bind complexed polyP retained in the protein network after paraformaldehyde fixation.

We then took the opposite approach and increased polyP levels by incubating cells with exogenous polyP chains, which are routinely taken up by mammalian cells. These experiments revealed that increasing the levels of polyP in cells did indeed increase cisplatin-mediated toxicity in a chain length-dependent manner. While the polyP-mediated augmentation of cisplatin toxicity was highly reproducible and significant, the relative increase in cytotoxicity was considered modest and ranged between 5 and 10%. However, we would like to emphasize that supplementation of exogenous, synthetic polyP is an imperfect approach to modify intracellular polyP distribution and/or abundance. In fact, when we monitored the subcellular localization of fluorescently-labeled polyP_300_ chains, it was evident that they did not fully recapitulate the nucleolar localization of endogenous polyP fractions (Figure S4D). Hence, we expect much more pronounced enhancement of cisplatin potency by polyP once we will be able to genetically manipulate endogenous polyP levels. Nevertheless, our results are supported by published reports, which showed that supplementation of lung cancer cells with exogenous polyP increased radiation-induced DNA double strand breaks and decreased cell survival (47). Moreover, the pro-metastatic protein h-Prune, whose expression correlates with lung cancer progression, has been shown to be a short-chain exopolyphosphatase *in vitro* (19, 48). Taken together, these findings suggest that mammalian cancer cells might have acquired mechanisms to regulate their cellular polyP levels and hence their drug resistance. Therefore, a strategy to increase polyP levels might be a promising avenue in the attempt to sensitize cancer cells to cisplatin treatment. This, however, requires the ultimate breakthrough in polyP research, namely the discovery of the mammalian polyP regulatory machineries and the development of pharmacological tools to target them efficiently and specifically.

## Data Availability Statement

The datasets generated for this study are available on request to the corresponding author.

## Author Contributions

LX, UJ, and DK contributed to the conception and the design of the study. LX, AR, NT, JE, ASR, BS, MM, and DK acquired and analyzed data for this work. EQ contributed to data analysis. EQ and DK performed the statistical analysis. DK wrote the first draft of the manuscript. LX and EQ wrote sections of the manuscript. All authors contributed to manuscript revision, read and approved the submitted version.

### Conflict of Interest

The authors declare that the research was conducted in the absence of any commercial or financial relationships that could be construed as a potential conflict of interest.
