# Supplementary material for: Accumulation of Nucleolar Inorganic Polyphosphate Is a Cellular Response to Cisplatin-Induced Apoptosis
Source: Front Oncol. 2019 Dec 12;9:1410. doi: 10.3389/fonc.2019.01410 (PMC6920253; doi:10.3389/fonc.2019.01410)
Supplement: Supplementary file 1 [file Table_1.DOCX]

Supplementary Materials
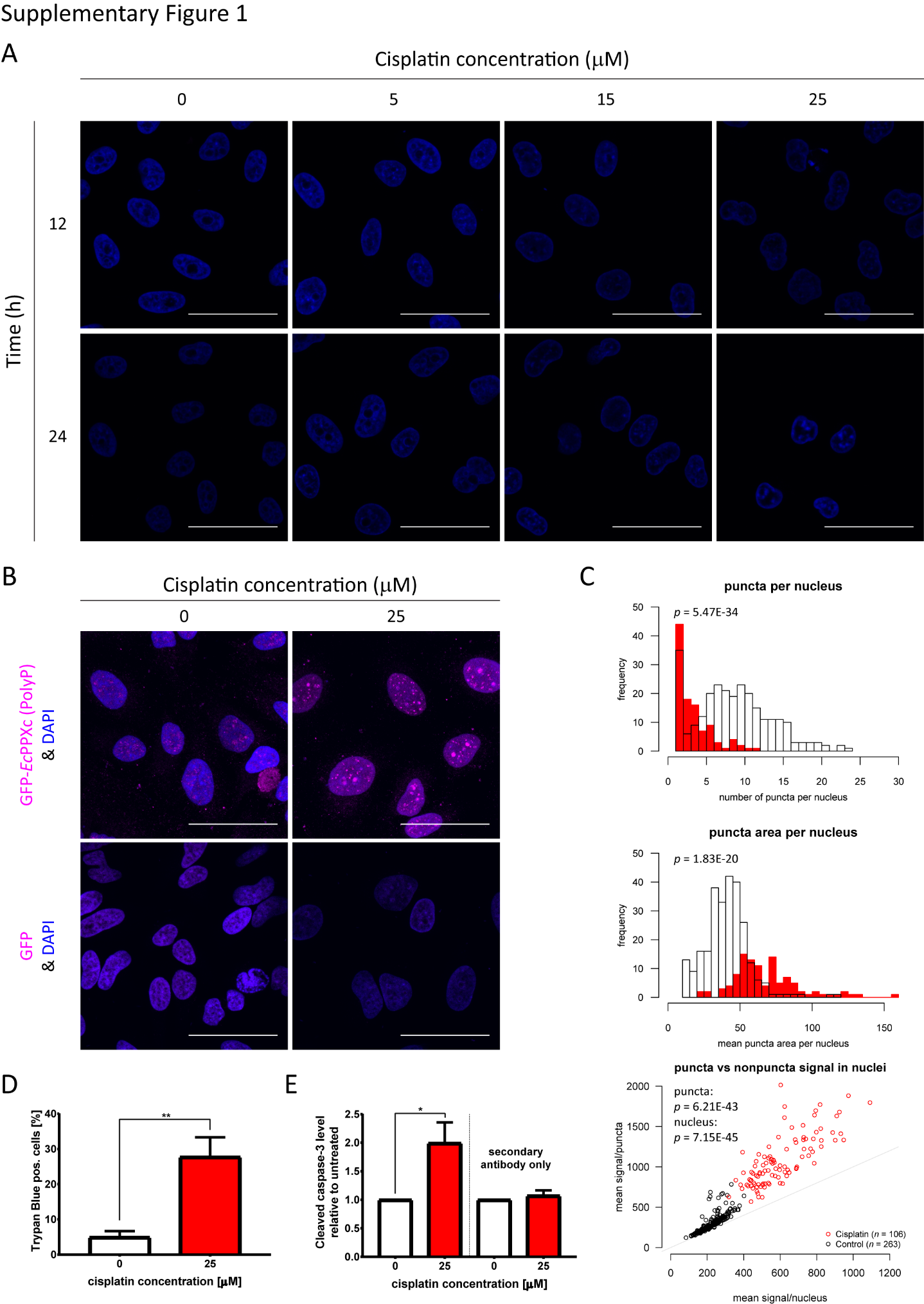


**Supplementary Figure 1.** **Cytotoxic cisplatin treatment causes polyP foci formation in cancer cells.**(**A**) Control images of HeLa cells labeled with GFP, instead of GFP-*Ec*PPXc, after various cisplatin treatments. Signal in the GFP channel (magenta) was negligible, indicating a very low level of unspecific binding of the GFP-*Ec*PPXc probe. DAPI (blue) was used to locate the cell nuclei. Representative images of projected z series are displayed. Scale bar: 50 μm. (**B**) PolyP accumulation and foci formation in HeLa-ATCC cells following cisplatin treatment. An overlay of GFP-*Ec*PPXc (magenta) and DAPI (blue) signals revealed the newly formed polyP foci in the nucleus of cisplatin-treated cells. This phenomenon is polyP-specific, as no such changes were observed with GFP labeling. Representative images of projected z series are displayed. Scale bar: 50 μm. (**C**) Quantification of polyP foci in untreated HeLa cells (white bars and symbols) and cells treated with 25 μM cisplatin for 24 hours (red bars and symbols). There is a change in the number (top panel), area (middle panel), and fluorescence intensity (bottom panel) of polyP foci upon cisplatin exposure. A representative analysis is shown. Welch Two Sample *t*-tests were performed, and the FDR-adjusted *p* values for the representative experiment are displayed. (**D**) Cisplatin-induced cell death measured by Trypan blue staining. (N = 7, mean + s.e.m., unpaired *t*-test, ***p* = 0.0021). (**E**) Apoptosis induction monitored by the increased levels of cleaved caspase-3 immunofluorescence. (N = 9, mean + s.e.m., unpaired *t*-test, **p* = 0.0148). No significant changes were observed in the unspecific binding of the fluorescent secondary antibody alone.


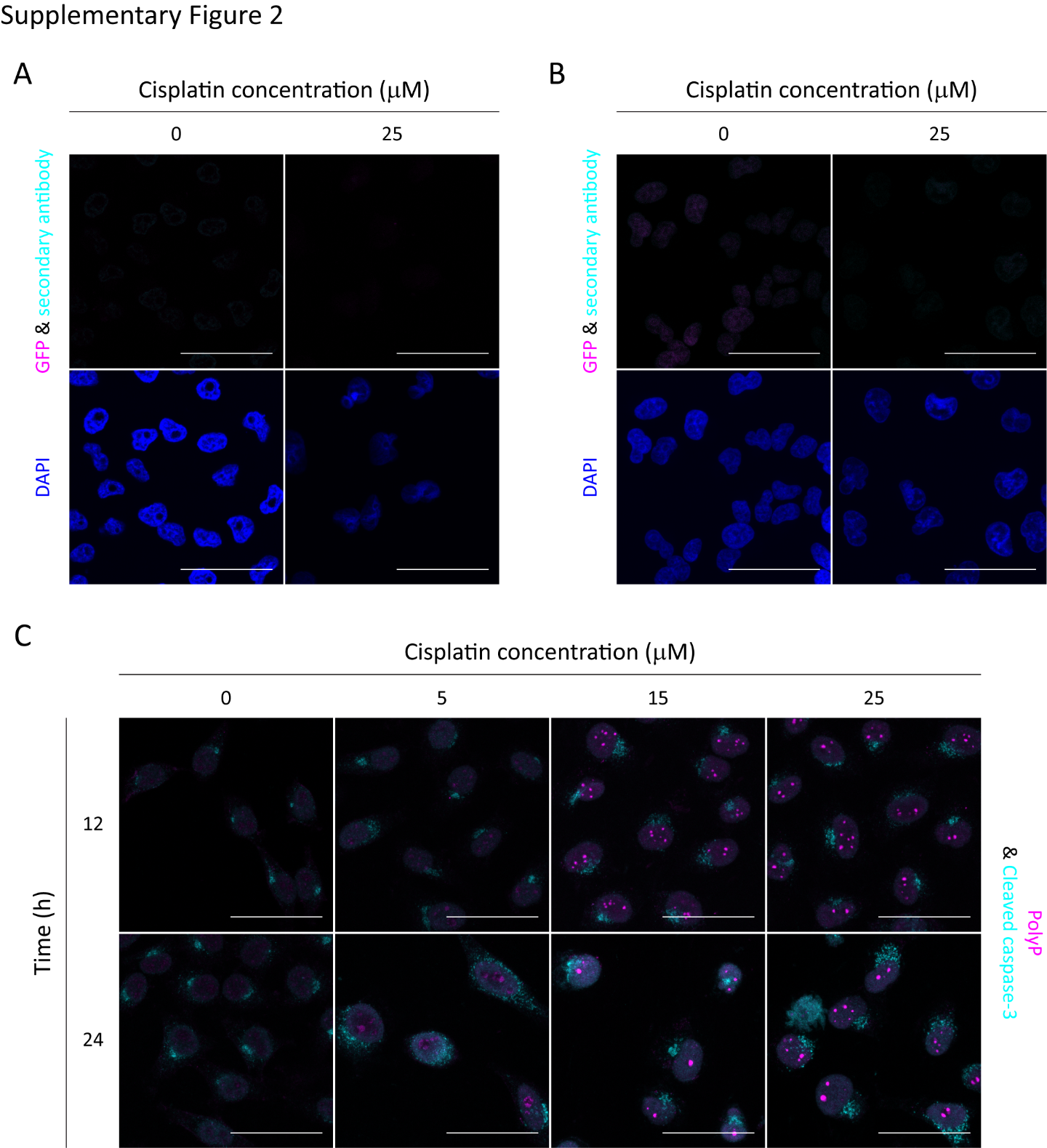


**Supplementary Figure 2.** **Cellular localization of polyP and cleaved caspase-3 after cisplatin treatment.**(**A** and **B**) HeLa cells, untreated or treated with 25 µM cisplatin for 24 hours, were labeled with GFP and the corresponding fluorescent secondary antibodies for (**A**) NPM1 and (**B**) RNA Pol I staining. An overlay of GFP (magenta) and the secondary antibody (cyan) signals is shown to assess the levels of unspecific binding. Nuclear DNA, illustrated by DAPI fluorescence, was used to locate the cells. Representative images of projected z series are displayed. Scale bar: 50 μm. (**C**) Co-staining using GFP-*Ec*PPXc (magenta) and a cleaved caspase-3 antibody (cyan) depicted the dose- and time-dependence of apoptosis induction in HeLa cells triggered by cisplatin, and revealed distinct localization of polyP and cleaved caspase-3 in the cells. Representative images of projected z series are displayed. Scale bar: 50 µm.


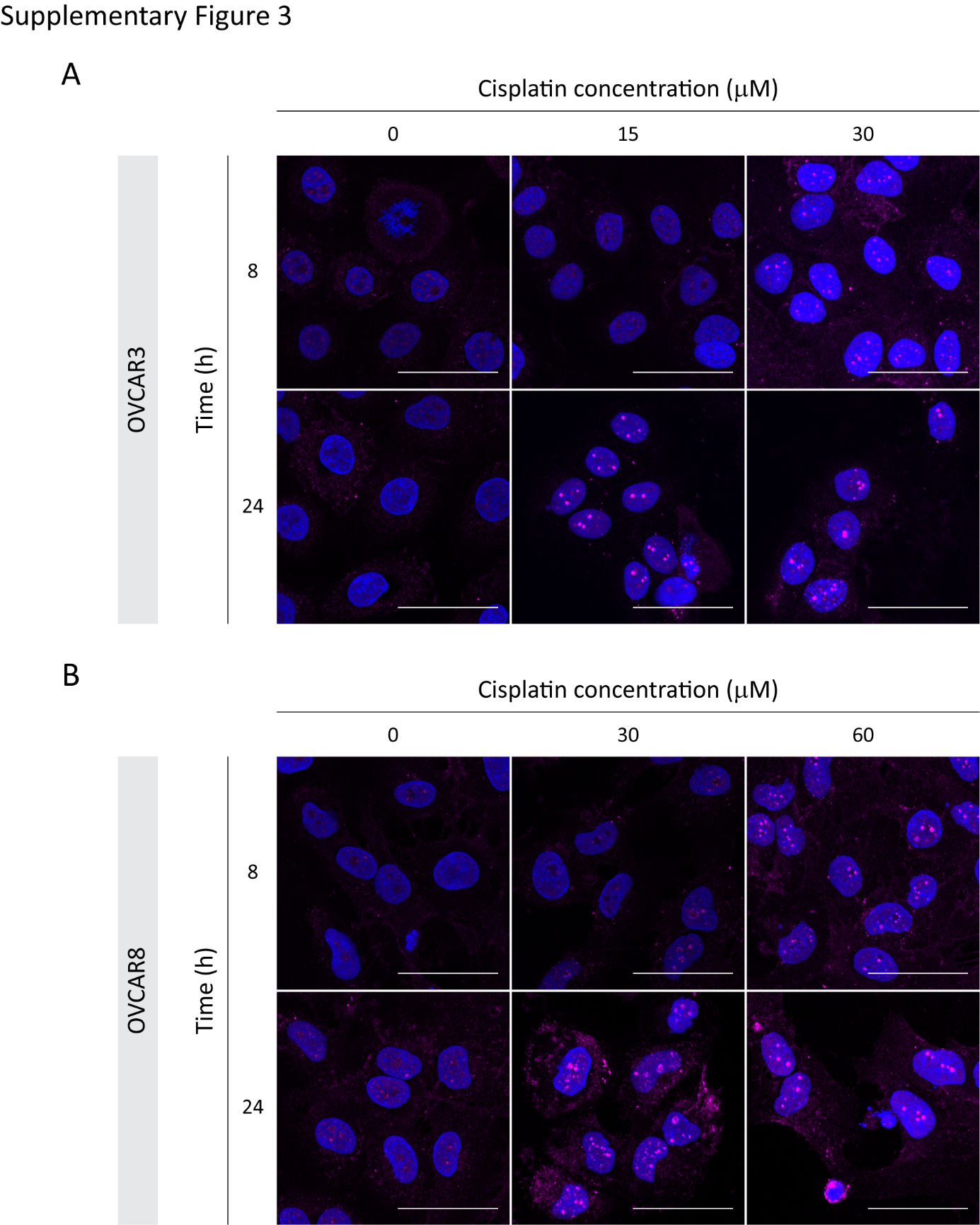


**Supplementary Figure 3.** **Dose- and time-dependence of cisplatin-induced polyP response in ovarian cancer cells.**(**A**) Cisplatin-sensitive ovarian cancer cell line OVCAR3 and (**B**) cisplatin-resistant ovarian cancer cell line OVCAR8 were treated with increasing concentrations of cisplatin for 8 and 24 hours and labeled with GFP-*Ec*PPXc (magenta) to monitor the changes in polyP levels and distribution. Nuclear DNA was revealed by DAPI fluorescence (blue). Representative images of projected z series are displayed. Scale bar: 50 μm.


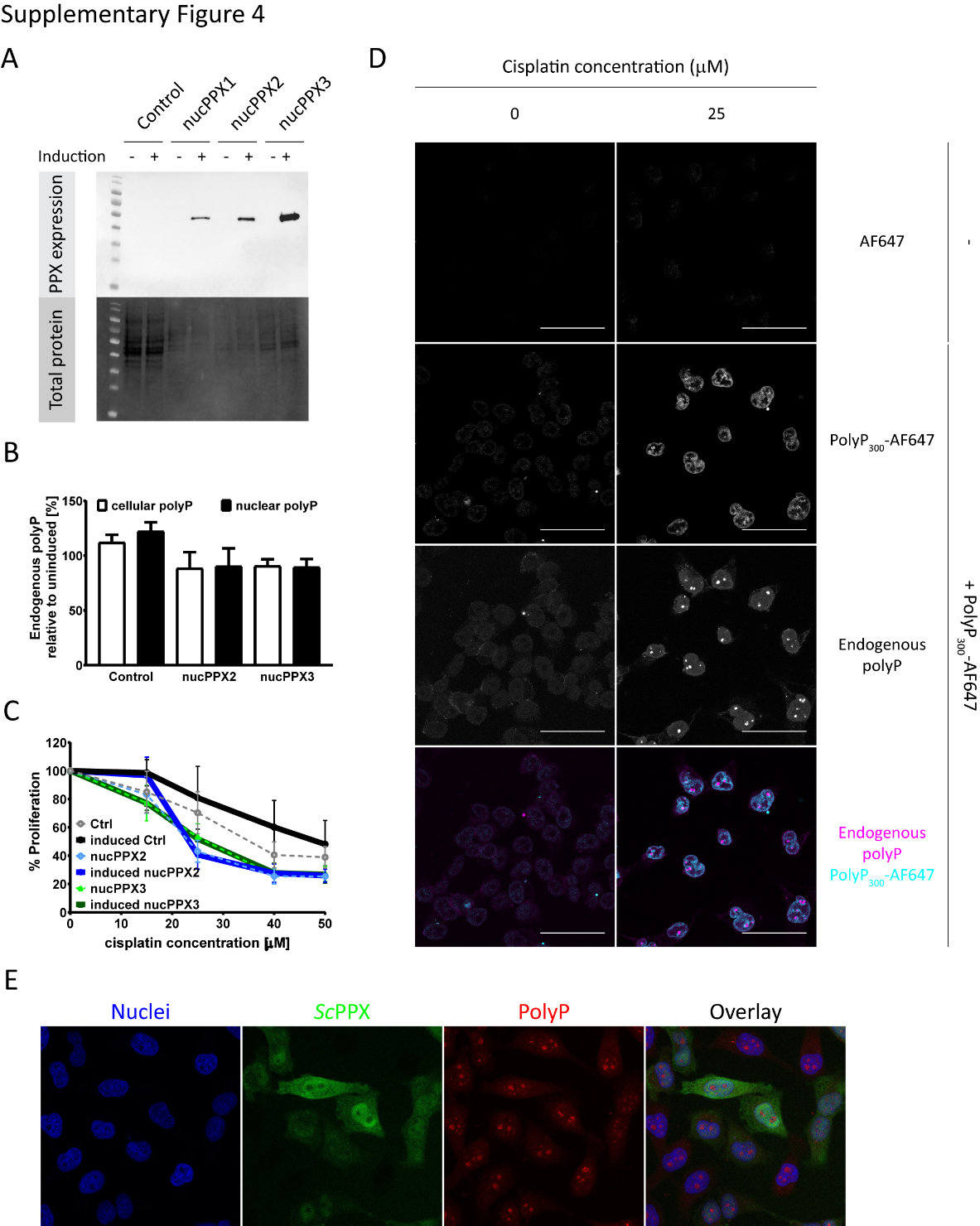


**Supplementary Figure 4.** **Manipulation of intracellular polyP levels and distribution in HeLa cells.**(**A**) Western Blot analysis of doxycycline-induced FLAG-tagged *Sc*PPX expression in wildtype HeLa cells (Control) and three independent clones of stably transfected HeLa cells with FLAG-tagged *Sc*PPX (nucPPX1-3). The signal of anti-FLAG antibody is shown in the top panel. The Bio-Rad Mini-PROTEAN® TGX Stain-Free™ protein gel allowed visualization of the amount of cell lysates run on the gel (bottom panel). (**B**) Quantification of endogenous polyP levels in the total cell (white bars) and in the nucleus (black bars) following the induction of *Sc*PPX expression. PolyP levels were normalized to the corresponding non-induced samples. The average and s.e.m. of 3 experiments for the wildtype control and two stable clones are shown. (The nucPPX1 clone did not show any effect in two biological replicates). (**C**) Proliferation of wildtype HeLa cells (black and gray) and *Sc*PPX stable clones (blue and green) upon cisplatin exposure. *Sc*PPX expression was induced by doxycycline (solid lines) and compared to the non-induced samples (dashed lines). The average of 4 experiments and the s.e.m. are displayed (The nucPPX1 clone has been tested twice and showed similar results). (**D**) Uptake and redistribution of fluorescently-labeled polyP in parallel to cisplatin treatment. 200 µM Alexa Fluor 647-labeled polyP₃₀₀ was supplemented to HeLa cells in the absence or presence of 25 μM cisplatin for 24 hours. The cellular localization of endogenous polyP (labeled with GFP-*Ec*PPXc, magenta) and exogenous polyP (Alexa Fluor 647-labeled, cyan) seemed to be different. (**E**) Subcellular localization of polyP and transiently-expressed *Sc*PPX in HeLa cells. An overlay of the fluorescence signal of anti-FLAG antibody (green), mCherry-*Ec*PPXc (red), and DAPI (blue) showed distinct compartmentalization of polyP and FLAG-tagged *Sc*PPX in the nucleus of HeLa cells.
